# Integrated Metabolomic and transcriptomic analyses reveal deoxycholic acid promotes transmissible gastroenteritis virus infection by inhibiting phosphorylation of NF-κB and STAT3

**DOI:** 10.1186/s12864-024-10167-8

**Published:** 2024-03-04

**Authors:** Yajing Zhou, Chao Xu, Shanshen Gu, Yeyi Xiao, Shenglong Wu, Haifei Wang, Wenbin Bao

**Affiliations:** 1 Key Laboratory for Animal Genetics, Breeding, Reproduction and Molecular Design, College of Animal Science and Technology, Yangzhou University, 225009 Yangzhou, China; 2 Joint International Research Laboratory of Agriculture and Agri-Product Safety, The Ministry of Education of China, Yangzhou University, 225009 Yangzhou, China

**Keywords:** TGEV, Metabolomics, Transcriptomics, Deoxycholic acid, Virus replication

## Abstract

**Background:**

Acute diarrhea, dehydration and death in piglets are all symptoms of transmissible gastroenteritis virus (TGEV), which results in significant financial losses in the pig industry. It is important to understand the pathogenesis and identify new antiviral targets by revealing the metabolic interactions between TGEV and host cells.

**Results:**

We performed metabolomic and transcriptomic analyses of swine testicular cells infected with TGEV. A total of 1339 differential metabolites and 206 differentially expressed genes were detected post TEGV infection. The differentially expressed genes were significantly enriched in the HIF-1 signaling pathway and PI3K-Akt signaling. Integrated analysis of differentially expressed genes and differential metabolites indicated that they were significantly enriched in the metabolic processes such as nucleotide metabolism, biosynthesis of cofactors and purine metabolism. In addition, the results showed that most of the detected metabolites involved in the bile secretion was downregulated during TGEV infection. Furthermore, exogenous addition of key metabolite deoxycholic acid (DCA) significantly enhanced TGEV replication by NF-κB and STAT3 signal pathways.

**Conclusions:**

We identified a significant metabolite, DCA, related to TGEV replication. It added TGEV replication in host cells by inhibiting phosphorylation of NF-κB and STAT3. This study provided novel insights into the metabolomic and transcriptomic alterations related to TGEV infection and revealed potential molecular and metabolic targets for the regulation of TGEV infection.

**Supplementary Information:**

The online version contains supplementary material available at 10.1186/s12864-024-10167-8.

## Background

A highly contagious intestinal disease swine transmissible gastroenteritis (TGE) is brought on by the transmissible gastroenteritis virus (TGEV). After being discovered for the first time in the United States in 1946, it quickly rose to one of the most common viral diarrhea in pigs [1]. TGEV target to the intestinal epithelium of pigs through fecal-oral route. In addition, TGEV is similar to SARS-CoV and SARS-CoV-2, multiplies in the nasal mucosa and then spreads through the bloodstream to the target organs [2]. TGEV belongs to the genus Coronaviridae of the Coronavirus family [3]. In addition to 5 non-structural proteins, the TGEV genome also encodes 4 structural proteins, including spike glycoprotein, envelope protein, membrane glycoprotein, and nucleocapsid protein [4]. In suckling piglets, TGEV is characterized by severe watery diarrhea, vomiting, and dehydration, with a high mortality rate of up to 100% [1]. However, there is no effective therapy available for TGE. Viruses promote anabolism for generation of macromolecules depending on host cell machinery to propagate, and viral infection activates metabolic reprogramming to facilitate optimal virus production [5]. Viruses can affect the metabolic phenotypes such as the increased nucleotide and lipid biosynthesis to meet the demands of virus infection [6]. Different viruses have different tactics to infect host cells and result in distinct metabolic landscapes [7].

During the virus infection, metabolites and metabolic pathways undergo important modifications. Zhou et al. found that during hepatitis B virus infection, glycolysis was activated to impede the production of interferon induced with retinoic acid-inducible gene Ι [8]. Li et al. have reported that glucose metabolism was generated to facilitate the biosynthesis of precursors required for Japanese encephalitis virus replication [9]. Thus, metabolomic analysis offers an effective and new insight on how to evaluate bioactive compounds and metabolic processes [10]. The comprehensive analysis of metabolome data and other omics data, such as transcriptome and proteome, is a potent method for detecting and characterizing metabolites and molecular mechanisms underlying biological phenotype [11]. Several physiological processes, such as cell division and apoptosis are impacted by modifications in cellular metabolism [12]. At present, it is unclear how TGEV interacts with the metabolism of host cells during the infection.

In this study, we used ST cells as a study model and we performed metabolomics and transcriptomics analyses to discover bioactive compounds, signaling pathways and biosynthetic regulatory genes during TGEV infection. A collection of differential metabolites and differentially expressed genes associated with TGEV infection were identified and the key metabolite DCA was found to play important roles in promoting TGEV infection. The results provided the metabolomic and transcriptomic landscape of host cells upon TEGV infection and contributed to our further understanding of TGEV pathogenesis.

## Results

### Identification of differential metabolites in TGEV-infected ST cells

**Fig. 1:**
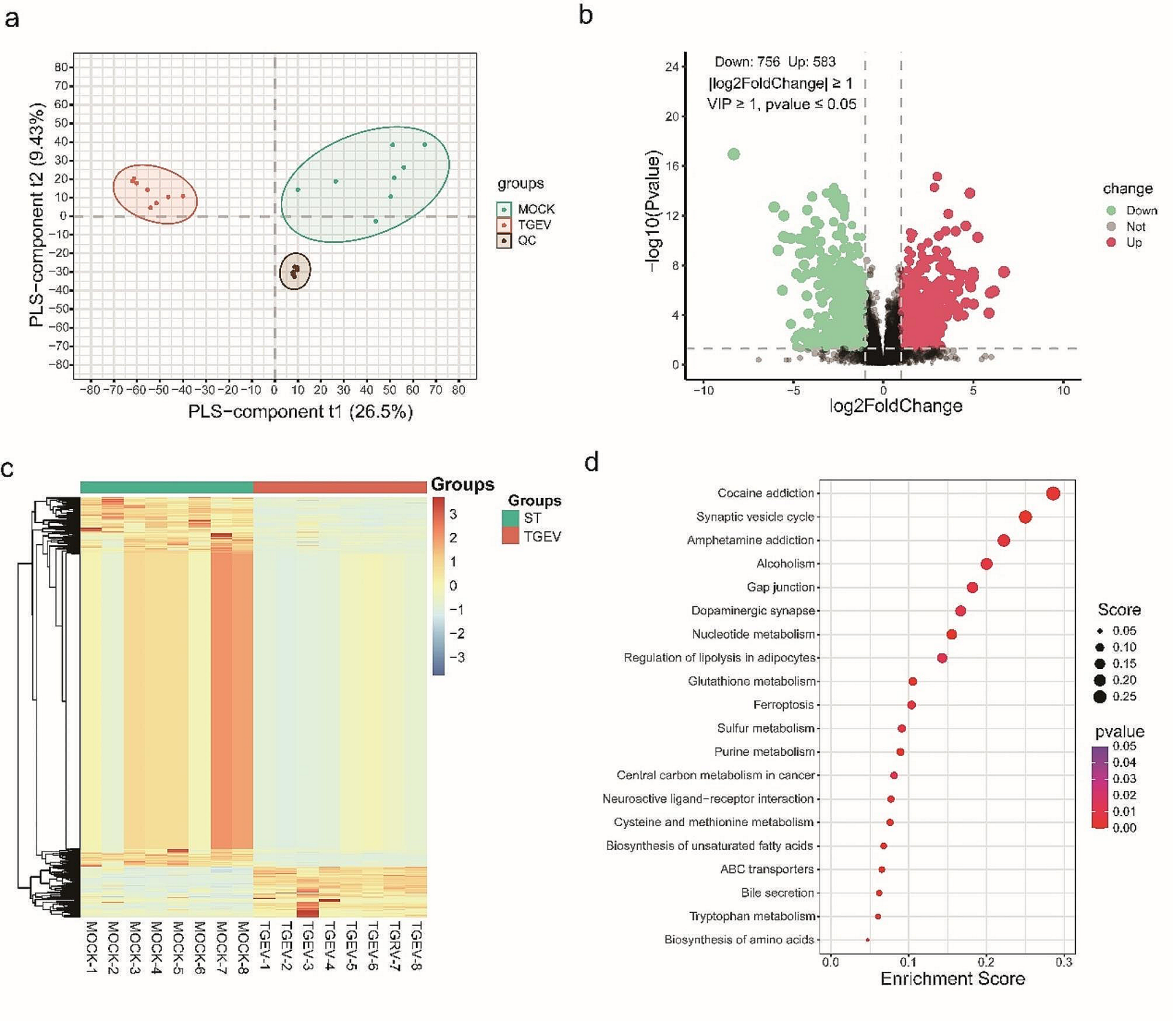
Metabolomics analysis of cells infected with TGEV. (**a**) Cluster analysis of the samples based on the identified metabolites using partial least-squares approach. Representing the TGEV-infected group and the mock group, respectively, are red and green dots. QC: quality control samples. (**b**) Volcano plot showing the differential metabolites between the two groups. Significantly down-regulated and up-regulated metabolites are indicated by green and red dots, respectively. (**c**) Hierarchical clustering analysis of the samples using the differential metabolites. Blue to red colors represent metabolites expression levels. (**d**) Pathways enriched by the different metabolites

To explore the metabolites involved in the cellular responses to TGEV infection, eight TGEV-infected cell samples and eight uninfected control samples were collected for metabolomics analysis. The partial least-squares discrimination analysis clearly separated the samples into two groups, which indicates the significant effects of TGEV infection on host cell metabolome (Fig. 1a). A quality control sample (QC) was prepared from a mixture of sample extracts to analyze the repeatability of the sample infected or not infected with TGEV. Differential metabolite analysis revealed a total of 1339 differential metabolites, with 756 down-regulated metabolites and 583 up-regulated metabolites (Fig. 1b, Table S2). The hierarchical clustering further describes the expression patterns of the differential metabolites between the two groups (Fig. 1c). To uncover the potential metabolic pathways associated with TGEV infection, KEGG enrichment was performed for the differential metabolites. The results showed that the pathways including nucleotide metabolism, purine metabolism, ABC transporters and bile secretion were significantly enriched (Fig. 1d, Table S3).

### Transcriptomics analysis of TGEV infected ST cells

**Fig. 2:**
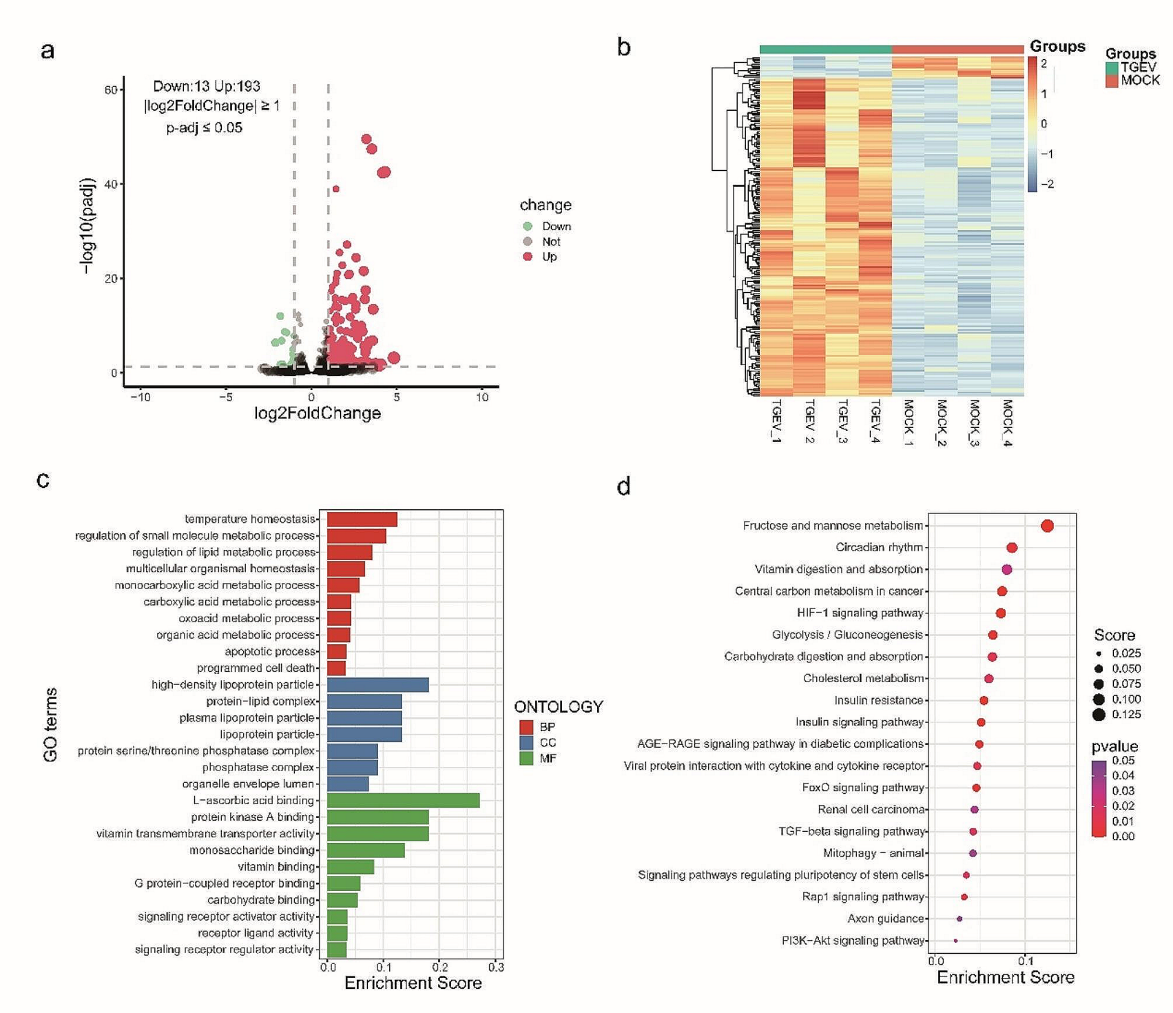
Transcriptomics analysis in TGEV-infected and mock ST cells. (**a**) Volcano plots of differentially expressed genes between TGEV-infected and mock groups. Significantly down-regulated and up-regulated genes are denoted by green and red dots, respectively. (**b**) Hierarchical clustering analysis between the two groups based on the differentially expressed genes. Blue to red colors represent gene expression levels. (**c**) Gene ontology (GO) analysis of differentially expressed genes. BP: biological process, CC: cell component, MF: molecular function. (**d**) KEGG pathway analysis of differentially expressed genes

To elucidate the potential regulators involved in the cellular reactions to TGEV infection, cell samples infected with TGEV and mock were collected for transcriptome analysis using RNA-seq. Differential expression analysis revealed 206 differentially expressed genes, of which 13 were down-regulated and 193 were up-regulated (Fig. 2a, Table S4). To display the expression patterns of the two groups, hierarchical clustering analysis was carried out based on the differently expressed genes (Fig. 2b). Gene ontology analysis indicated that the differentially expressed genes were categorized into 27 subcategories, including ‘regulation of small molecule metabolic process (GO:0062012)’, ‘monocarboxylic acid metabolic process (GO:0032787)’, and ‘carboxylic acid metabolic process (GO:0019752)’ (Fig. 2c, Table S5). The pathway enrichment analysis revealed that the HIF-1 signaling pathway, the PI3K-Akt signaling, and the insulin signaling pathway were significantly enriched by the differentially expressed genes (Fig. 2d, Table S6).

### Integrated analysis of metabolome and transcriptome data

**Fig. 3:**
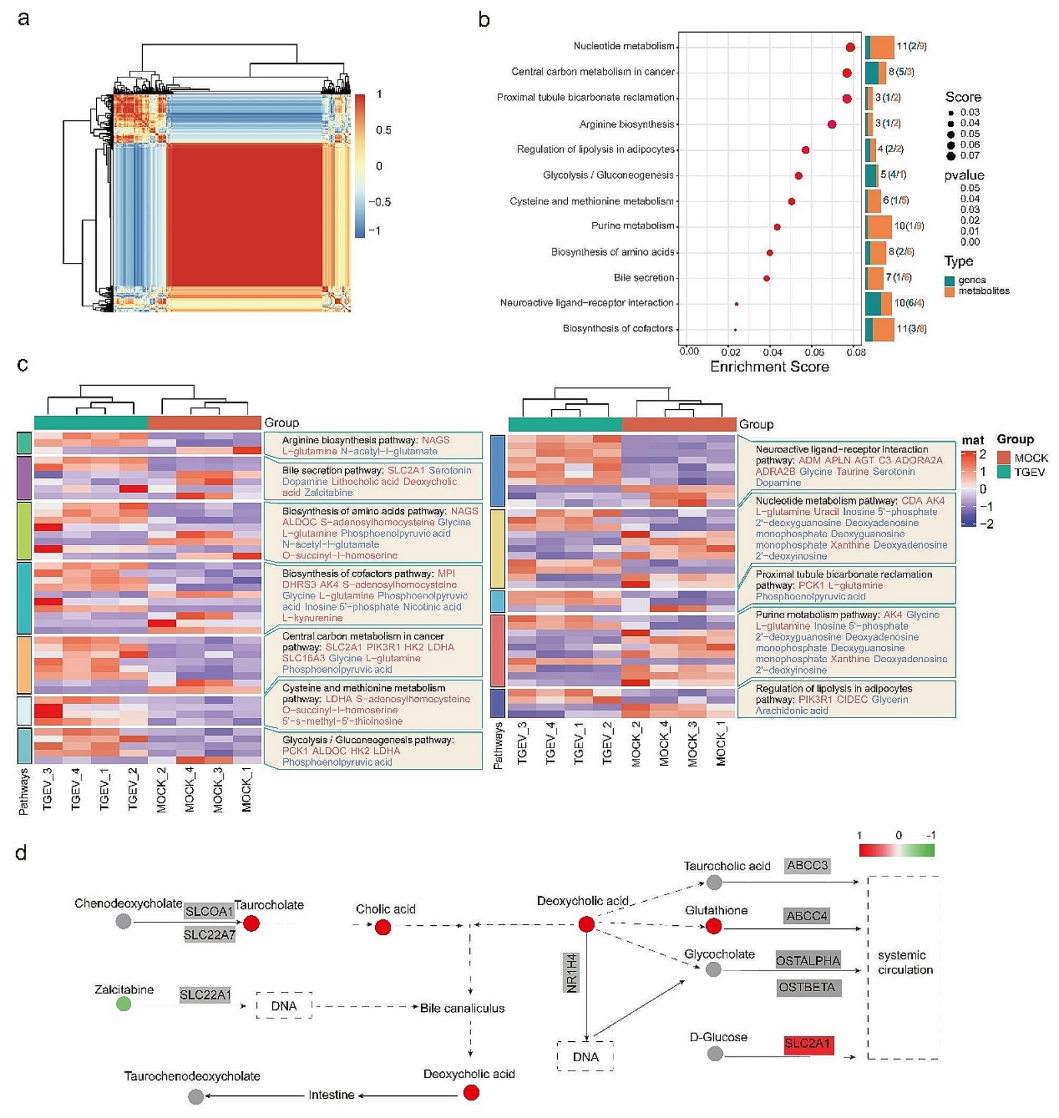
Integrated analysis of differential metabolites and differentially expressed genes between the TGEV-infected and mock groups. (**a**) Hierarchical clustering of the differential metabolites and differentially expressed genes. Blue to red colors represent metabolites and genes expression levels. (**b**) Pathway analysis for differential metabolites and differentially expressed genes. (**c**) The list of DEGs and metabolites dysregulated post-TGEV infection. (**d**) Metabolites and genes involved in bile secretion. The up-regulated and down-regulated metabolites are indicated by red and green dots, accordingly. The undetected metabolites are indicated by gray dots. The grading colors represent changes of gene expression

An integrated analysis of differential metabolites and differentially expressed genes were carried out to investigate the molecules and signaling pathways connected to TGEV infection. Hierarchical clustering analysis showed that the correlation of differential metabolites and differentially expressed genes between the two groups (Fig. 3a). We the conducted pathway enrichment and found that the metabolites and genes were significantly enriched in the metabolic processes including nucleotide metabolism, biosynthesis of cofactors and purine metabolism (Fig. 3b, Table S7). Among the enriched pathways, bile secretion was also dramatically affected in which most of the detected metabolites were down-regulated after TGEV infection (Fig. 3c and d, Table S8), indicating the potential roles of bile secretion in the regulation of TGEV infection.

### Deoxycholic acid increases TGEV replication in ST cells by inhibiting NF-κB and STAT3 signal pathways

**Fig. 4:**
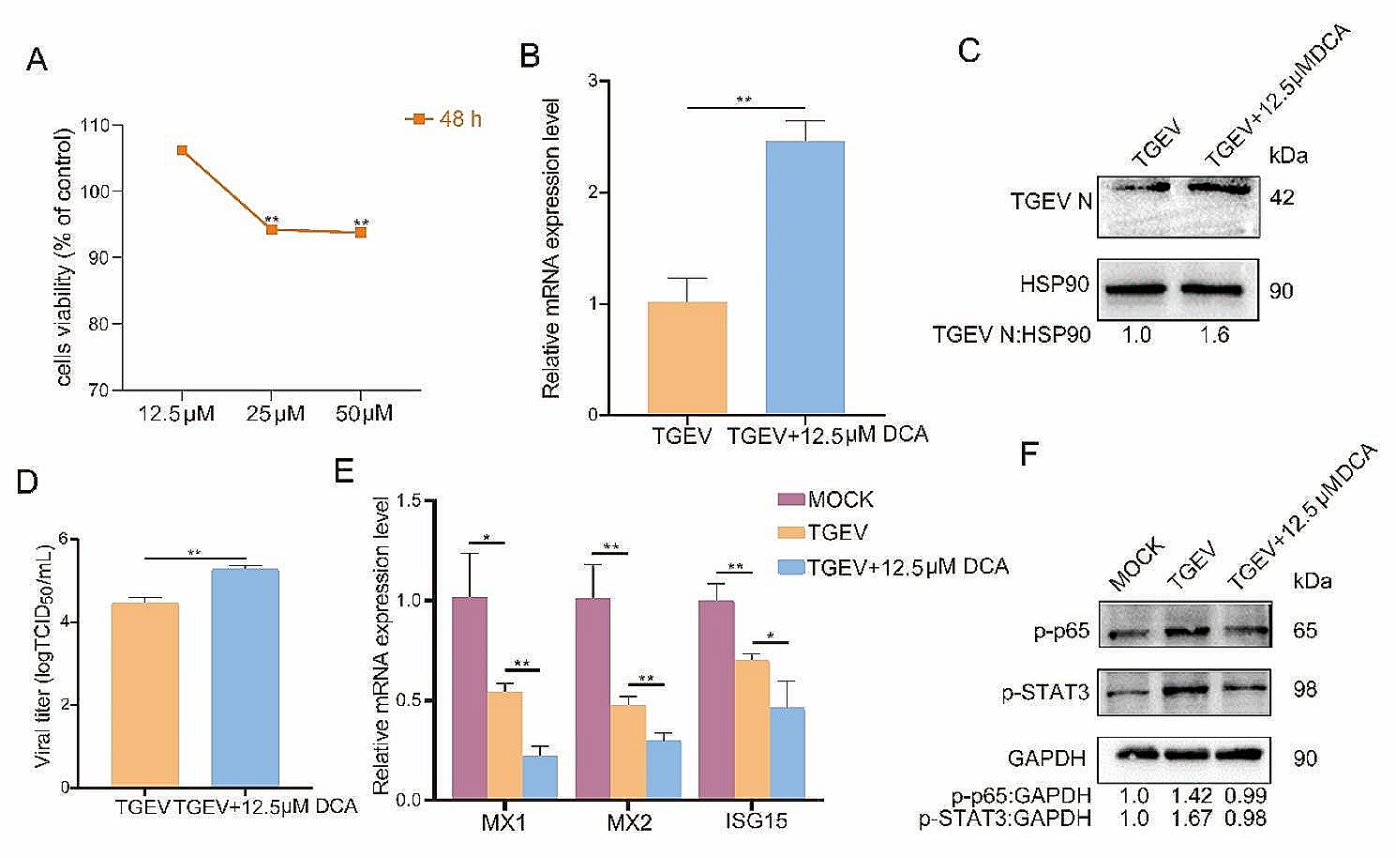
Effects of DCA addition on PEDV replication in ST cells. (**a**) Viability of ST cells treated for 48 h with DCA at various doses (12.5 µM, 25 µM, 50µM). (**b**) Relative expression of TGEV N gene after 48 h of TGEV infection. The results are represented as the mean ± SD of three separate tests (*n* = 3). ^**^P<0.01. (**c**) Western blot of TGEV-N protein. (**d**) The titer of TGEV treated with DCA after 48 h. (**e**) Relative expression of antiviral genes. (**f**) Western blot of p-p65 and p-STAT3 protein. MOCK: ST cells; TGEV: ST cells infected with TGEV; TGEV + 12.5 µM DCA: ST cells infected with TGEV and treated with DCA.

Deoxycholic acid (DCA) is involved in resisting the invasion of pathogens into host cells [13]. To explore whether DCA regulates TGEV infection, we quantified the copy numbers and protein expression of TGEV in TGEV-infected ST cells. We first determined the suitable dose of DCA in ST cells using CCK8 assay and found that 12.5 µM DCA has no obvious effects on the viability of ST cells at 48 h (Fig. 4a). We then investigated the effects of DCA addition on TGEV replication in ST cells. qPCR analysis showed that DCA addition significantly increases the expression of TGEV N gene (Fig. 4b). Furthermore, western blot analysis confirmed the increased TGEV N protein level in ST cells with DCA addition (Fig. 4c). TCID_50_ assay further demonstrated that DCA addition significantly increased the titer of TGEV (Fig. 4d). In additional, during TGEV infection, the expression levels of antiviral genes MX1, MX2, and ISG15 were significantly downregulated upon DCA addition (Fig. 4e).

It was reported that NF-κB activation occurred in TGEV infected ST cells by affecting p65 phosphorylation [14]. Furthermore, activation of STAT3 signal pathway suppressed the infection of TGEV [15]. To investigate the mechanism through which DCA promotes the infection of TGEV, we tested the effects of DCA on NF-κB and STAT3 phosphorylation. The results showed that DCA addition lowered TGEV-induced phosphorylation of NF-κB and STAT3(Fig. 4f). Together, these results suggested that DCA increases the replication of TGEV in host cells by inhibiting phosphorylation of NF-κB and STAT3.

## Discussion

It is well known that different viruses adopt different strategies to complete viral replication by hijacking host metabolism [16]. Metabolomics analysis is an effective method to identify the metabolites involved in virus infection. For example, the Newcastle disease virus infection induced modifications to the metabolism of amino acids and nucleotides [17]. Central carbon metabolism, especially glycolysis changed significantly altered during dengue virus infection [18]. In addition, the host amino acid metabolism was significantly changed during African swine fever virus infection [19]. Bile acid plays a key role in resisting hepatitis B virus infection via controlling the activation and metabolism of T cells [20]. Therefore, it is an important antiviral strategy to explore the mechanism of various virus regulating the metabolism of host cells. Herein, metabolomics analyses were performed to detect metabolites with significant alterations in TGEV infection. There was significant change in bile secretion, it reflects the intracellular role in understanding how TGEV controls host metabolism to facilitate reproduction. Our work offers a new insight into the interactions between TGEV infection and host cells.

Transcriptome is a high-throughput approach to study the interactions between host cells and virus. In this study, KEGG analyses revealed that differential expression genes mainly involved in cellular signaling pathways including HIF-1 signaling pathway, PI3K-Akt signaling pathway and insulin signaling pathway. The antiviral response of host cells mainly depends on the activation or inhibition of different signaling pathways. For instance, hypoxia inducible factor-1α is activated to induce the production of pro-inflammatory cytokines during virus infection [21]. The PI3K/AKT signaling pathway is activated to impair the cell growth and survival and then induce virus replication during dengue virus infection [22]. Therefore, the results indicated the important functions of these pathways in defense of TGEV infection. Besides, we found that solute carrier family 2 member 1 (SLC2A1) was up-regulated in TGEV infection and involved in bile secretion. SLC2A1 plays an important role in metabolism and is associated with immune infiltration [23]. It is reported that increasing the expression of SLC2A1 would suppress epstein-barr virus replication [24]. Thus, the study indicated that SLC2A1 is a key gene involved in the replication of TGEV.

Bile acid metabolism was associated with the development of numerous illnesses, including intestinal hepatitis, infection, tumorigenesis and diabetes [25]. In addition, bile acid plays different immunoregulatory activity on the innate and adaptive immune systems, including promoting inflammation in macrophages and the regulatory effect on T cell differentiation [26]. However, the immune regulation mechanism of bile acid is complicated due to its diversity of structure and biological activity. For example, the activation of TGR5-Camp-PKA axis by bile acid limits the activation of NLRP3 inflammatory and IL-1β to block the inflammatory reaction of monocytes [27]. Besides, the evidence showed that viral infection can promote the innate antiviral immune response of different cell types to induce the accumulation of bile acids through biosynthesis and absorption [28]. DCA, which belongs to the secondary bile acid metabolites, can convert unconjugated primary bile acids to secondary bile acids and regulate host metabolism via G-coupled protein receptor TGR5 [29–30]. Hao et al. found that bile acids, especially DCA, activate signals 1 and 2 of NLRP3 inflammatory corpuscles in macrophages differentiated under cholestasis or sepsis, but they are not activated in a dose-dependent manner in monocytes [31]. Herein, we revealed that the differential metabolites associated with TGEV infection were involved in bile secretion. DCA addition obviously increases the replication of TGEV in host cells.

The findings showed that bile secretion may be important metabolic processes for TGEV infection in ST cells. In the early stages of infection, the metabolic activity of the virus is crucial for evading the host’s innate immune defenses and the replication and proliferation of virus [32]. The relationship between the metabolite and gene expression has been researched in cell lines via integrating the transcriptomic and metabolomic [33]. Ortmayr et al. found that there was a relationship between the transcriptional regulation activity of fifty-three cell lines and metabolites [34]. At present, metabolomics can be used as a technique to study the prevention of viral infection [35]. It suggested that the genome has a broad influence on the regulation of metabolites. Herein, our research utilized the integration of metabolomics and transcriptomics to explore the metabolic features of ST cells during TGEV infection, and we found that the metabolites and differential genes were enriched in nucleotide metabolism, purine metabolism and central carbon metabolism in cancer. Viruses can utilize the nucleotide metabolism in host cells to facilitate virus replication. For example, herpes simplex virus I encoded ribonucleotide reductase and thymidine kinase to create an environment benefited to its replication [36]. Epstein-Barr virus induced the virus replication through activating the progress of purine and pyrimidine biosynthesis [37]. Besides, central carbon metabolism in cancer meditating the replication of Epstein-Barr virus had also been reported [38]. Therefore, we inferred that nucleotide metabolism and central carbon metabolism in cancer are involved in TGEV replication, but it requires further investigation.

## Conclusions

Together, our study systematically showed the metabolomics and transcriptomics landscapes of TGEV infection in host cells. Genes and metabolites with significant changes were identified. Integrated analysis revealed the most affected metabolic processes including nucleotide metabolism, biosynthesis of cofactors and purine metabolism. Functional investigations indicated the roles of DCA in adding the replication of TGEV in host cells by inhibiting phosphorylation of NF-κB and STAT3. The findings will further the understandings of the interactions between TGEV and host cells and reveal potential metabolic and molecular targets to prevent and control TGEV infection.

## Materials and methods

### Cells and virus

The ST cells were purchased from China Center for Type Culture Collection (Wuhan, China). The Dulbecco’s Modified Eagle’s Medium (DMEM, Gibco, Grand Island, USA) which contains 10% fetal bovine serum (FBS, Gibco, Grand Island, USA) and 1% penicillin/streptomycin (Solarbio, Beijing, China) was used to cultivate the cells. TGEV were preserved in our laboratory and propagated in ST cells cultured with DMEM and 2% FBS.

### Cell sample preparation

ST cells were grown overnight under the condition of 37 ℃ with 5% CO_2_ after being seeded in six-well plates at a density of 1 × 10^5^/well. TGEV was introduced into the test wells at a MOI = 0.1. After 48 h of treatment, TGEV-infected and uninfected control cells were collected, promptly frozen in liquid nitrogen, and then preserved at -80 °C for further use.

### Metabolomics analysis

Eight biological replicates in TGEV infected ST cells or mock were set up for metabolomics detection. BGI-Shenzhen (Shenzhen, China) supplied metabolites extraction and LC-MS/MS analysis. The Waters UPLC I-Class Plus (Waters, USA) tandom Q Exactive high resolution mass spectrometer (Thermo Fisher Scientific, USA) was used for the separation and detection of metabolites. Peak alignment and metabolite quantification of the original data were performed using Compound Discoverer 3.1 software. Peak intensities were converted to total spectral intensity before being normalized. The peaks were matched to the mzCloud, mzVault, and Masslist databases (Thermo Fisher Scientific, Waltham) in order to provide precise and relative quantitative results. The MetaX was used to transform and normalize the data [39]. The KEGG (https://www.genome.jp/kegg/pathway.html), HMDB (https://hmdb.ca/) and LIPIDMaps (https://www.lipidmaps.org/) databases were utilized to generate the annotated metabolites. The expressed metabolites were deemed to have significantly changed at the level of VIP ≥ 1, *P* ≤ 0.05 and|log2-fold change| ≥ 1 between the two groups.

### Transcriptome data analysis

Four biological replicates in TGEV infected ST cells or mock were prepared. Total RNA was extracted using Trizol (Takara Biotechnology (Dalian), Dalian, Chian), and transcriptome sequencing on Illumina HiSeq-PE150 by Novo Gene Technology Co., Ltd. (Beijing, China). After eliminating low mass readings containing the adapter or readings with a base mass fraction lower than 20, clean reads were obtained. Genes were deemed to be expressed with fragments per kilobase million (FPKM) value was over than 1. The TopHat2 was used to count the clean reads refer to the Sscrofa11.1 genome [40]. The DESeq2 software was employed to detect the differentially expressed genes between both the two groups [41]. Genes were regarded to be differentially expressed with the threshold of|log2-fold change| ≥ 1 and adjusted *P* ≤ 0.05.

### Integrated network analysis of transcriptomics and metabolomics

Differentially expressed genes and metabolites were analyzed by Pearson correlation analysis. Common pathways had been identified after all of these genes and metabolites were uploaded to the KEGG pathway database (https://www.kegg.jp/kegg/pathway.html) [42–44]. According to the same pathways, the biological and signal transduction pathways connected to the differentially expressed genes and metabolites were identified.

### Cell viability assay

ST cells were contained with DMEM and planted in 96-well plates at a density of 4 × 10^3^/well. After overnight incubation, the cell culture medium was replaced with DMEM containing various amounts of deoxycholic acid (DCA, MCE, NJ, USA) (12.5 µM, 25 µM, 50 µM). Each well was added with 10 µL of Cell Counting Kit-8 (Yeasen, Shanghai, China) solution and the Tecan Infinite 200 microplate reader (Tecan, Switzerland) was employed to measure the results at 450 nm.

### qPCR assay

Total RNA from cells were isolated by Trizol (Takara Biotechnology (Dalian), Dalian, Chian), which was then used as the template for reverse transcription. HiScript III RT SuperMix for qPCR (Vazyme, Nanjing, China) was utilized to synthesize cDNA in accordance with the manufacturer’s instructions. Utilizing AceQ Universal SYBR qPCR Master Mix (Vazyme, Nanjing, China), qPCR was performed. The system (10 µL) contained 1 µL template of cDNA, 5 µL of 2 × AceQ Universal SYBR qPCR Master Mix, 0.2 µL of each primer and 3.6 µL of deionized water. The following were the reaction conditions: 95 ℃ for 5 m, 40 cycles of 95 ℃ for 10 s and 60 ℃ for 30 s. The level of mRNA expression was normalized with GAPDH. The sequence of primers used in the qPCR assays are shown in Table S1.

### Western blot analysis

Cells were washed three times with PBS and collected with RIPA lysis buffer containing protease inhibitor cocktail. The titer of protein was quantified using Enhanced BCA Protein Assay Kit (Beyotime, Shanghai, China). SDS-PAGE was utilized to separate whole cell lysates after they had been denatured for 10 min in 5×SDS-PAGE loading buffer. At room temperature for 2 h, the proteins were transferred to the polyvinylidene fluoride membranes sealed with TBS containing 5% skimmed milk powder and 0.2% Tween 20. The anti-TGEV-N (DA0224, Youlong Biotech, Shanghai, China), anti-phospho-NF-κB p65 Ser 536 (#3033, Cell Signaling Technology, Danvers, MA, USA), anti-phospho-STAT3 S727 (CY5291, Abways Techology, Beijing, China)and anti-HSP90 (60318-1-Ig, Proteintech, Wuhan, China) antibodies were incubated with the membranes at 4 ℃ overnight. The membranes were then treated with the goat-anti-mouse IgG (1:5000) or goat-anti-rabbit IgG (1:5000) secondary antibodies for 2 h at room temperature. The proteins were detected by enhanced chemiluminescence (Thermo Fisher Scientific).

### 50% cell culture infectious dose (TCID_50_) assay

ST cells were seeded in 96-well plates at a density of 10^4^/well in 100 µL of DMEM containing 10%FBS and 1% penicillin/streptomycin. The virus was then serially diluted (1:9, 10 µL into 90 µL) across the plate from columns 1 to 6, with pipette tips changed between each column. The plates were then incubated at 37 ℃ with 5% CO_2_ for 7 days. A Reed and Muench calculation was the performed to determine the 50% infectious dose, which was then scaled up to give a count of the TCID_50_ per mL [45].

### Statistical analysis

All data are presented as mean ± standard derivation (SD). Student’s t-test was used to compare the differences between different groups using GraphPad Prism. P-values<0.05 were considered statistically significant (^*^P<0.05, ^**^P<0.01).

### Electronic supplementary material

Below is the link to the electronic supplementary material.

Supplementary Material 1

Supplementary Material 2

Supplementary Material 3

Supplementary Material 4

Supplementary Material 5

Supplementary Material 6

Supplementary Material 7

Supplementary Material 8

Supplementary Material 9

## Data Availability

The RNAseq data used in this study was obtained from the National Center for Biotechnology Information (NCBI) Gene Expression Omnibus (GEO) database under accession number PRJNA766131.The study includes the original contributions, which are presented in the article as well as in the Supplementary Material. The metabolomics data from the study are stored in the publicly accessible National Genomics Data Center (NGDC, https://ngdc.cncb.ac.cn/) database as accession number OMIX005818.
