# Supplementary figures and images for: Integrated Metabolomic and transcriptomic analyses reveal deoxycholic acid promotes transmissible gastroenteritis virus infection by inhibiting phosphorylation of NF-κB and STAT3
Source: BMC Genomics. 2024 Mar 4;25:239. doi: 10.1186/s12864-024-10167-8 (PMC10913532; doi:10.1186/s12864-024-10167-8)

Fig. 4c


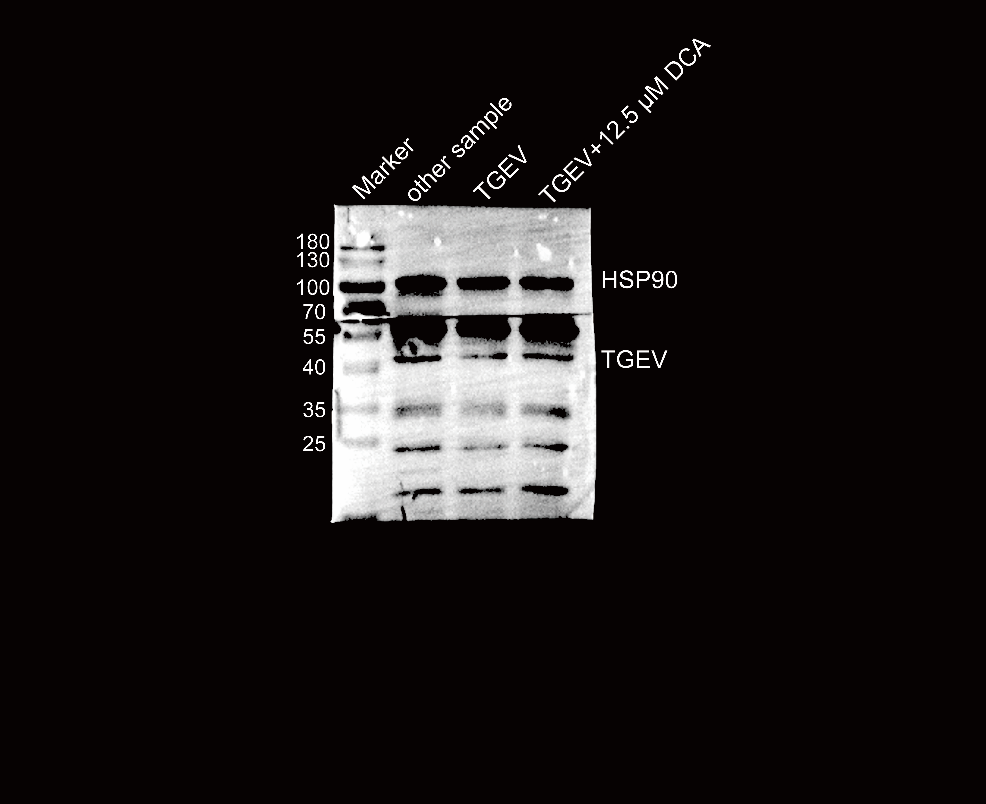


Fig. 4f


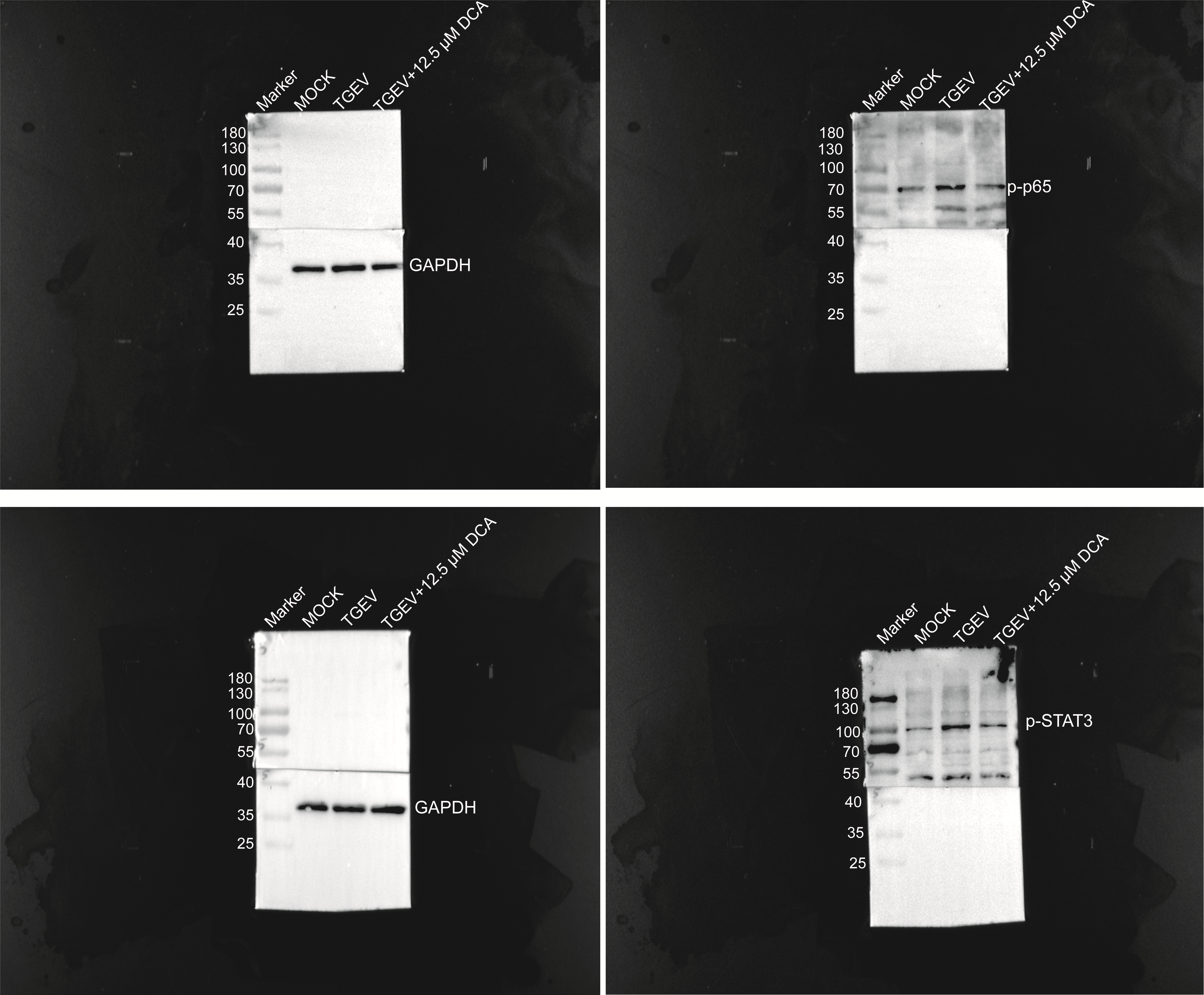

Supplement: Supplementary file 9 — Supplementary Material 9 [file 12864_2024_10167_MOESM9_ESM.docx]
